# TiN Plasmonic Metamaterial Arrays Fabricated at Low Temperatures on Versatile Substrates

**DOI:** 10.1021/acsaom.5c00449

**Published:** 2025-12-08

**Authors:** Ryan Bower, Daniel A L Loch, Ethan Muir, Bruno Rente, Xiaofei Xiao, Ming Fu, Papken Eh. Hovsepian, Arutiun P. Ehiasarian, Rupert Oulton, Peter K. Petrov

**Affiliations:** † Department of Materials, Royal School of Mines, 4615 Imperial College London, Exhibition Road, South Kensington, London SW7 2AZ, U.K.; ‡ Department of Physics, Blackett Laboratory, 4615 Imperial College London, Prince Consort Road, South Kensington, London SW7 2BW, U.K.; § National HIPIMS Technology Centre, Materials and Engineering Research Institute, 7314 Sheffield Hallam University, Sheffield S1 1WB, U.K.

**Keywords:** HIPIMS, plasmonics, nanolithography, titanium nitride, polymer substrates

## Abstract

Transition metal nitrides (TMNs) have emerged as promising alternative materials for plasmonic and optoelectronic applications in the visible and near-IR spectral ranges. However, the deposition of TMNs with optical properties suitable for plasmonic applications typically requires high temperatures (>800 °C). In this work, we use high-power impulse magnetron sputtering (HIPIMS) to deposit plasmonic TiN thin films at room temperature, without intentional heating. HIPIMS increases the energies of metal ions and dissociated nitrogen (N^1+^) in the flux, enabling the low-temperature deposition of high-quality TiN thin films on a range of industrially relevant substrates, including flexible polymer substrates. We demonstrate that the room-temperature deposition method can be used to produce plasmonic TiN nanoarrays via colloidal lithography, with tunable shapes and dimensions, creating features on the order of 100–500 nm. We characterize the optical response and plasmonic performance of these nanoarrays, demonstrating tailorable resonances in the visible and NIR spectral range, in agreement with optical simulations reported here.

## Graphical abstract

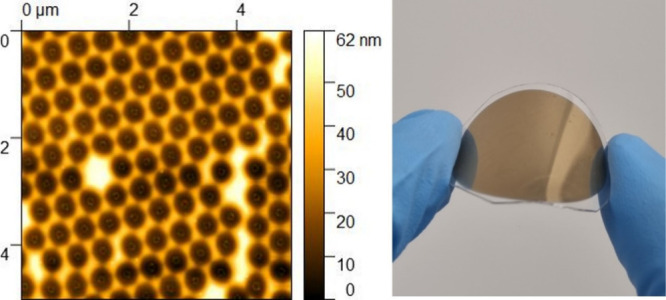

## Introduction

Transition metal nitrides (TMNs), such as titanium nitride, zirconium nitride, niobium nitride, and hafnium nitride,^1–5^ are promising for plasmonic applications including energy harvesting, catalysis, biological sensing, and thermoplasmonics.^6–11^ TMNs have several merits specific to each of these applications. For example, they are refractory materials that are mechanically and chemically robust, therefore lending themselves to certain operations within high temperature and corrosive environments.^12–17^ Several TMNs display optical properties comparable to the noble metals gold and silver and have therefore been highlighted for plasmonic applications such as sensing, absorption enhancement and hot electron generation.^5,18–20^

Furthermore, when considering TMNs for plasmonic applications, the tailorability of their optical properties with stoichiometry has been highlighted as a potential advantage. TMN stoichiometry can be controlled either by cation substitution and the production of ternary and quaternary transition metal nitrides or by varying the nitrogen content.^21–24^ Both methods alter the charge carrier concentration, shifting the plasma frequency. The optical response of TMNs can also be modified by altering the deposition parameters including temperature, pressure, substrate bias, and substrate-target distance.^25–27^ This control of plasmonic response can allow for the production of plasmonic devices with tailorable resonances and enhancements, broadening the potential applicability of these materials while retaining a smaller footprint.

TMNs have also been suggested as alternatives to noble metals for the fabrication of CMOS devices incorporating plasmonic components. The most widely studied plasmonic materials, Au and Ag, are incompatible with CMOS fabrication processes and devices.^28^ One solution is to use alternative plasmonic materials that are readily compatible with CMOS fabrication methods, including TMOs, TMNs, and metals.^29^ Several TMNs are also compatible with Si photonics and integrated circuit design, with TiN used as a gate material in CMOS chips.^30,31^

There remain several barriers to the widespread application and integration of TMNs within functional devices^32^ often arising from the elevated temperatures (>700 °C) required for the deposition of high-quality plasmonic TMN thin films. These high deposition temperatures have restricted the integration of TMN plasmonic components within CMOS devices and fabrication processes, which typically require deposition temperatures of <400 °C.

The production of nanoscale TMN components is also hampered by the difficulty encountered in patterning these mechanically robust materials. Top-down patterning techniques, which use ion milling or reactive ion etching, require high powers, sacrificial hard masks, and often, the use of harsh fluorine and chlorine-based chemicals.^18,33–35^ While this is suitable for some applications, alternative fabrication methods with fewer process steps are desirable. Lift-off is a bottom-up alternative to top-down nanofabrication techniques where the material of choice is deposited through a sacrificial mask that is subsequently removed, thereby yielding a nanostructured array while wholly omitting the etching step. Typically, lift-off relies upon deposition through a polymer mask, so the application of this nanofabrication method to TMN thin films has also been restricted by the high deposition temperatures required for the fabrication of plasmonic TMN thin films.

Indeed, the low-temperature deposition of TMNs has been the subject of a significant amount of research in recent years. Various physical vapor deposition methods have been demonstrated to enable the low-temperature deposition of TMN thin films including pulsed laser deposition (PLD),^36^ plasma enhanced atomic layer deposition (PE-ALD),^34,37^ electron-beam evaporation,^38^ reactive magnetron sputtering,^35,39–41^ HIPIMS,^42–44^ and R-HIPIMS with glancing angle deposition (GLAD).^45^ This has led to the production of CMOS-compatible refractive index sensors,^46^ temperature sensors,^47^ and photodetectors.^48^ Evidently, the successful low-temperature deposition of transition metal nitride thin films has the potential to introduce an additional degree of flexibility when considering both nanofabrication of functional devices and integration into device fabrication processes, especially for devices with complex architectures.

We describe a room-temperature deposition method using HIPIMS suitable for the deposition of TiN thin films onto industrially relevant substrates, including polymers. Furthermore, we successfully fabricated titanium nitride metamaterial arrays via selective area deposition. Thin films of TiN are deposited using HIPIMS onto a sacrificial polymer mask, and subsequent lift-off enables the preparation of TMN nanohole arrays at ambient temperature. We demonstrate that the combination of room-temperature TiN deposition with the colloidal lithography nanofabrication method used herein facilitates the scalable fabrication of metamaterial arrays with a tailorable optical response.

## Results and Discussion

### 1 Low- and Room-Temperature Deposition of TMN Thin Films Using HIPIMS

#### Results

Titanium nitride thin films were deposited at room temperature using HIPIMS. Deposition conditions are outlined in the Experimental Methods, and further details including example HIPIMS pulse parameters and characteristics of the ion flux to the substrates during deposition are included in Supplementary Section 1. The films were deposited to a total thickness of 100 nm onto a range of industrially relevant substrates: steel (mirror-polished stainless-steel grade 304; polishing was done using mechanical grinding and polishing using 1 μm diamond paste), soda-lime glass, and silicon (001 orientation, p-doped). In addition, thin films were also deposited onto flexible polymer substrates: polyethelyne naphthalate (PEN) and polyimide (PI). These flexible polymers have a range of applications within the aerospace, energy, automotive, and electronics industries.^49–51^ The fabrication of plasmonically decorated polymer substrates can yield low-cost, flexible plasmonic sensors.^52,53^

Figure 1 shows spectroscopic ellipsometry data collected for the TiN thin film samples deposited at room temperature and indicates that these films are optically metallic, as demonstrated by a negative real part of the dielectric permittivity, (ε’). The crossover wavelength, where the real dielectric permittivity changes sign, is similar for all films and lies within a range of 20 at ∼475 nm. This wavelength correlates with the screened plasma frequency ω_sp_ for the metal films and is dependent upon the charge carrier concentration and therefore the stoichiometry of the films, with a consistent ω_sp_ indicative of stoichiometric titanium nitride films.^54^

**Figure 1:**
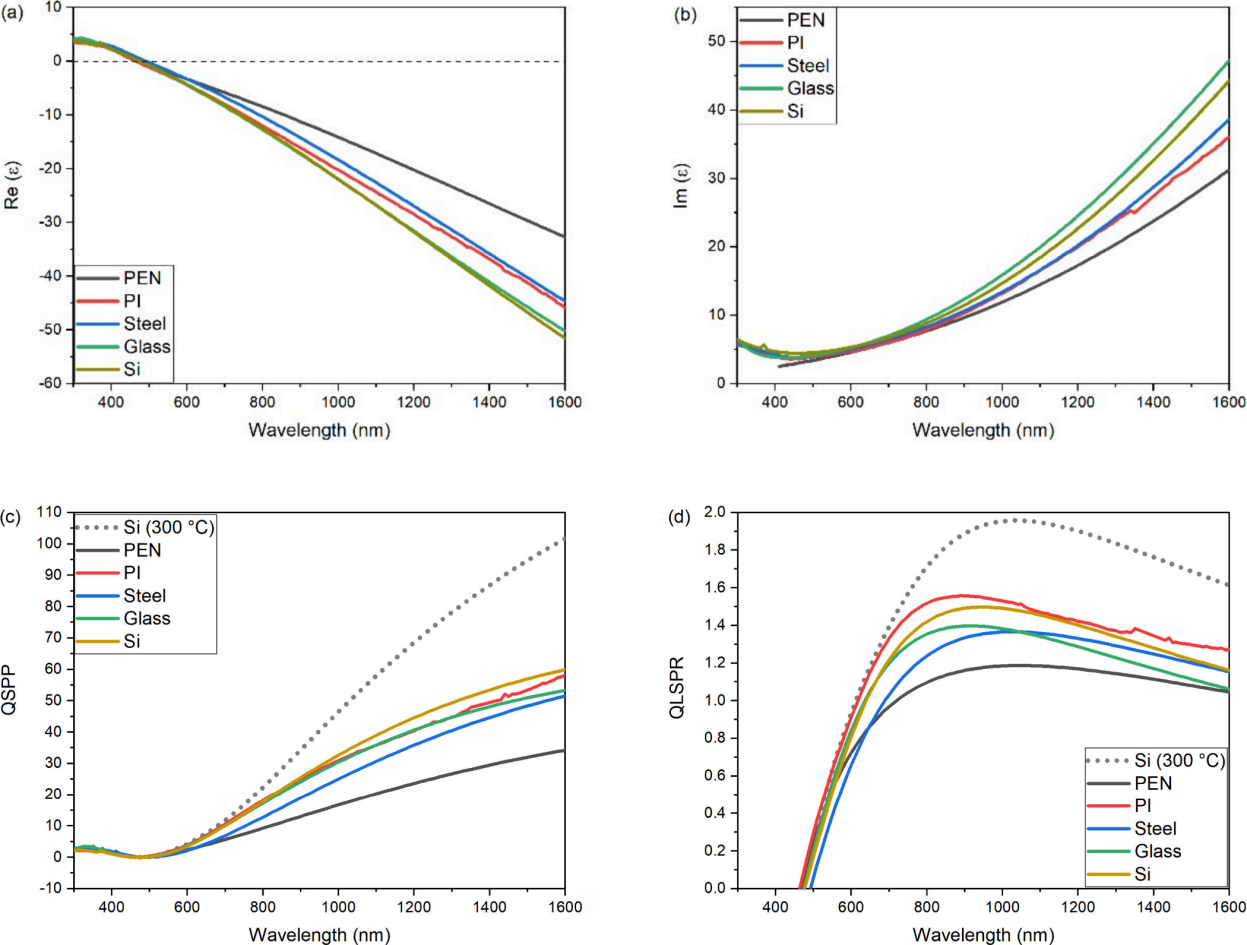
Real (a) and imaginary (b) dielectric permittivity data for 100 nm TiN films deposited at room temperature, extracted from spectroscopic ellipsometry measurements. (c) Surface plasmon resonance figure of merit (*Q*_SPP_) and (d) localized surface plasmon resonance (*Q*_LSPR_) figure of merit data for each of the TiN thin films deposited on various substrates and comparison to deposition at 300 °C on Si substrate.

There is some variation in the real dielectric function at longer wavelengths. This is further reflected in the differences in imaginary permittivity, ε”. The imaginary permittivity is closely related to the losses within the films and at longer wavelengths arises due to intraband transitions within TiN, that is, absorption of light by free carriers. Typically, metallic films display higher losses because of these intraband absorptions when compared to dielectric thin films. In metals, the carrier concentration, defect density, grain size, and resultant scattering will all affect the magnitude of intraband absorptions within the films. As the crossover wavelength of the TiN thin films is consistent, the scattering magnitude is likely to be a result of variations in crystallinity and grain size within the thin films arising from strain and lattice mismatch with the substrates.

XRD data were collected to assess the crystallinity of the thin films. All films are polycrystalline, as shown in Figure S3, displaying crystallographic peaks, which are characteristic of the TiN 111 and 002 lattice planes, respectively. However, the diffraction peaks have low intensity and are relatively broad, indicating poor crystallinity within the films. This is not unexpected for films deposited at room temperature and has previously been reported for low and room-temperature depositions of TiN thin films,^40^ whereas thin films deposited at higher deposition temperatures are known to display less amorphization. It should be noted that variations in crystal structure will affect the plasmonic performance, impacting the losses within the materials, as has been previously reported in the literature.^25,40,55,56^

In addition to the breadth of the XRD peaks, there is also a slight shifting of the central peak position for each of the different substrates. Variations in diffraction peak position and breadth can be influenced by the stoichiometry, including the extent of oxygen incorporation within the thin films, grain size, and strain, which can be a substrate-mediated effect. Chamber conditioning serves to minimize the incorporation of oxygen within the films, although trace amounts are present in the vacuum base pressure, as shown in the residual gas analyzer data collected during deposition (Figure S1). The shift in the diffraction peak position is likely to be dominated by the influence of grain size and substrate-mediated strain. This is consistent with the ellipsometry data previously discussed.

The grain size, strain, and degree of amorphization within the thin films could be further controlled by modifying the deposition parameters, including pressure and HIPIMS pulse parameters: peak power density, pulse length, and duty cycle. HIPIMS pulse parameters are known to alter the composition of the plasma flux, with increased peak power density yielding an increase in dissociation of nitrogen and ionization of Ti.^57^ In the conditions of the current experiment, the ion flux comprised a dominant fraction of metal ions of Ti^1+^ and Ti^2+^, while nitrogen ions were mostly in a dissociated state with N^1+^: N_2_^+^ ratio of 4.7 as shown in Figure S2. The same figure shows that the energy of metal and dissociated nitrogen ions, N^1+^, was elevated compared to the process gas ions of Ar^1+^ and N_2_^+^ as a result of the sputtering cascade. The high Ti^2+^:Ti^1+^ ratio of 1:2 is indicative of a high ionization degree of the metal vapor. The elevated ion energies and increased presence of metal ions with single and especially double charge in the sputtered species flux within the HIPIMS process, in contrast to DC and RF magnetron sputtering processes, are responsible for increased adatom mobility. This contributes to more homogeneous nucleation, dense grain boundaries, and larger grains from the outset of the growth. Control of the plasma composition within a HIPIMS process can therefore yield variations in the film composition and quality. For example, Ehiasarian et al. demonstrated that increasing peak current density and the subsequently high surface adatom mobility resulted in full morphological densification of TiN thin films^58^ while Yang et al. demonstrated that increased peak power density increased the magnitude of the real part of the dielectric permittivity for transition metal nitride thin films.^43^

To review the suitability of the TiN thin films for plasmonic applications, one can assess the plasmonic figures of merit, *Q*_SPP_ and *Q*_LSPR_ ^59^ extracted from the optical data. *Q*_LSPR_ is the quality factor for localized surface plasmon resonance (LSPR) and *Q*_SPP_ is the quality factor for surface plasmon polaritons (SPP), where *Q*_LSPR_ = −Re­(ε)/Im­(ε) and *Q*_SPP_ = Re­(ε)^2^/Im­(ε). These figures of merit give an approximate guide to the viability of a material for plasmonic applications. Notably, and as discussed by Lalisse et al. and Doiron et al, the figures of merit are not universal and can be further modified to consider properties more relevant to targeted applications.^5,60^ However, as a first benchmark to assess the quality of our TiN thin films deposited by using HIPIMS at room temperature, *Q*_SPP_ and *Q*_LSPR_ are sufficient. Figure 1c,d shows the figures of merit for our films deposited at room temperature. There is some variation in the figures of merit from films deposited on different substrates, likely arising from variations in crystallinity and grain size, as discussed above. Films deposited on PEN display the poorest performance compared to other, more standard substrates. When comparing the films deposited using HIPIMS at room temperature with those deposited at 300 °C in our previous work, we see that the higher temperature deposition does yield improved performance (dashed line in Figure 1c,d). This is not unexpected, as the higher temperature deposition will yield improved crystallinity of the films. Nevertheless, values obtained for these films are comparable to those previously reported in the literature as shown in Figure S4.^39^

We have therefore demonstrated that the deposition method and parameters selected for our TiN thin films yield polycrystalline metallic thin films at room temperature with optical properties comparable to those previously reported in the literature. We now investigate the combination of this deposition method with lift-off for the fabrication of nanostructured plasmonic metamaterial arrays.

### 2 Preparation of Nanostructured Arrays

The successful deposition of TiN thin films at room temperature enables etchless nanofabrication methods to be investigated, potentially simplifying the production of metamaterials arrays as used in plasmonic sensors, energy harvesting, and catalysis.^61,62^ We elected to use colloidal lithography to investigate room-temperature TiN deposition through a polymer mask. Colloidal lithography is a scalable nanofabrication technique, providing higher throughput than electron-beam lithography and improved feature resolution compared with standard photolithography processes. It has previously been demonstrated to be capable of providing large area coverage of nanoscale features up to the meter scale.^63,64^ The HIPIMS deposition process we have described could also be applied to lift-off using EBL and photolithography; however, as HIPIMS is an industrially scalable technique, we aimed to couple this with a similarly scalable nanofabrication method.

We investigated the fabrication of TMN nanohole arrays by depositing them through a sacrificial, self-assembled, colloidal mask. Hexagonally close-packed arrays of polystyrene (PS) nanospheres with an initial diameter of 500 nm were deposited onto Si and glass substrates using the “fishing” method, described in detail in the Experimental Methods. Following application of the colloidal mask, the diameter of the spheres comprising the sacrificial PS layer was then altered by reactive ion etching in an oxygen plasma, yielding PS diameters of 500, 460, 330, and 220 nm. Subsequently, a 100 nm thin film of TiN was deposited onto and through the PS mask by using the deposition method described above. Each sample was then characterized using SEM, AFM, and UV–vis spectroscopy before and after lift-off of the PS spheres. A witness sample was included in each deposition run as a control measure for the quality of the TiN thin films, with XRD and ellipsometry data from these films included in Supplementary Section 3c.

From the AFM and SEM micrographs, included in Figure 2 and Supplementary Section 3a,b, the HIPIMS deposition of TiN at room temperature is shown to successfully coat the PS mask and to yield a nanohole array upon lift-off of the mask. Notably, the PS spheres retain their morphology despite the energy dense plasma used during the HIPIMS deposition process. There is little variation in the PS structure after coating with TiN and the PS spheres retain their hexagonal arrangement. Some defects are visible in the micrographs, showing missing PS spheres and imperfect packing. These arise from incorrect mask application and are not introduced during the deposition process. HIPIMS is therefore a suitable method to deposit TiN thin films onto polymer colloids at room temperature.

**Figure 2:**
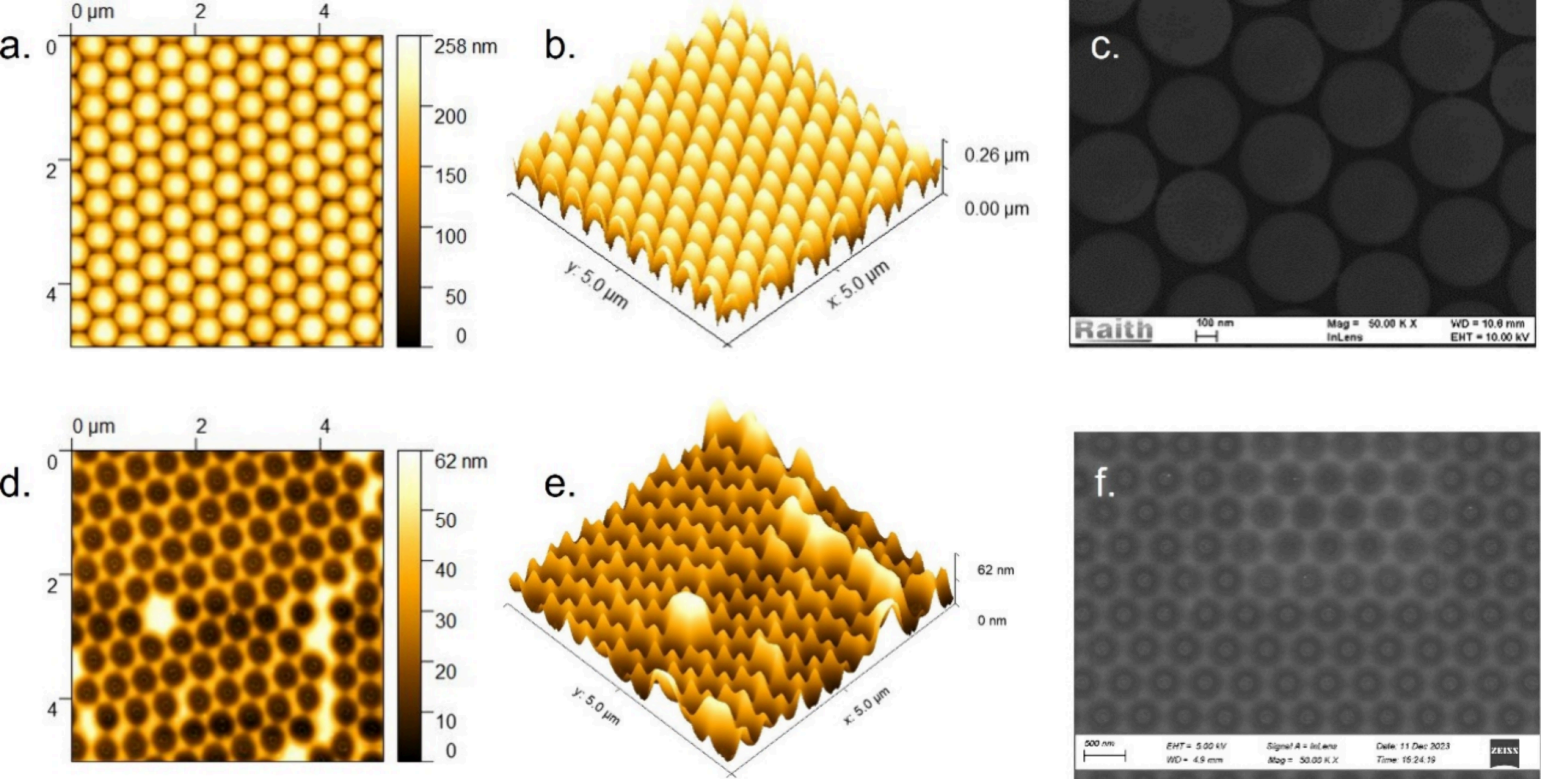
AFM (a, b) and SEM (c) micrographs of the PS mask exposed to the O_2_ plasma RIE for 15 s and then coated with 100 nm TiN. AFM (d, e) and SEM (f) micrographs of the same sample after lift-off of the PS colloidal mask.

Lift-off of the PS mask yields nanotriangular or nanohole arrays. The connectivity of the arrays can be controlled by altering the O_2_ plasma exposure time, with shorter etch times producing nanotriangle arrays as material is deposited in the interstices of the hcp PS array and longer etch times delivering nanohole arrays. The thickness of the arrays is less than 100 nm, as shown in the line profiles included in Supplementary Section 3b. Additionally, for nanohole arrays, the hole diameter does not exactly match the PS diameter. Due to the angled deposition of the sputtered materials and the elevated adatom mobility of the species deposited from the highly energetic plasma, the TiN thin film is also deposited underneath the PS mask. This shadowing effect has been highlighted as a limitation for the fabrication of plasmon-enhanced photovoltaics via colloidal lithography.^20^ These factors should be considered and further optimized when preparing nanohole arrays of various diameters, as required when targeting plasmon resonances at specific wavelengths for enhanced sensing applications.

HIPIMS offers a potential solution to the deposition directionality challenge mentioned above. Patsalas et al. previously suggested that increasing the directionality of the deposited sputter species was a method to successfully implement NSL for the fabrication of TiN nanoislands and demonstrated so by applying a substrate bias during deposition of a TiN thin film by DCMS.^61^ In contrast to DCMS, the high instantaneous powers applied during HIPIMS deposition processes result in a more energy dense plasma containing a high proportion of ionized metal species and dissociated nitrogen species, as discussed above.^57^ The momentum and direction of the charged species present within the plasma, including metal ions, can be directed by applying a substrate bias. Indeed, control of the applied bias during a HIPIMS deposition has previously been demonstrated to enable the homogeneous coating of high aspect ratio structures.^65^ Further investigation of the effect of substrate bias during the film deposition process could therefore be used to tailor the dimensions, morphology, and adhesion of the nanofeatures obtained when deposited through a colloidal mask. Additionally, the HIPIMS process enables the possibility of applying a bipolar HIPIMS pulse combined with a synchronized substrate bias pulse that could be used to modify the composition and morphology of the nitride thin films obtained, thereby tuning the optical properties of these plasmonic arrays.

We have successfully demonstrated that the room-temperature deposition method of TiN described above can be used to deposit TMN thin films onto and through a polymer mask. This represents a simplification of the manufacturing process for plasmonic arrays by removing the requirement for thin film etching. For all plasmonic applications, it is necessary to understand and tailor the optical response of the arrays, which we investigate further through UV–vis characterization.

### 3 Optical Characterization of Nanostructured Arrays

Figure 3a displays the normalized transmission spectra from TiN-coated PS spheres with diameters 500, 465, and 353 nm coated with 100 nm TiN and the bare TiN film. Transmission peaks are visible at approximately 400–600 nm, corresponding to the ENZ crossover region.^66^ At longer wavelengths, there are peaks and dips in transmission that arise due to plasmon resonances within the TiN meta-surface, more readily visible in the PS spheres that have undergone the shortest RIE time. The TiN caps deposited on the PS spheres exhibit an LSPR, while the TiN nanohole array displays coupled SPP and LSPR modes. The spectral features observed are not simply a combination of the TiN cap and TiN nanohole array spectra but will also include coupling between the modes of the TiN caps and the nanohole array, as previously reported in the literature for similar structures.^53,67,68^ These coupled modes will yield variations in transmission intensity observed. In general, for the samples measured, as the RIE etching time used for each sample prior to deposition increases and the feature diameter decreases, there is a blue shift in the wavelength of the peaks and dips in transmission. Typically, smaller features display a higher frequency resonance, and resonance frequency red-shifts as feature size increases. Additionally, the evolution of higher order quadrupolar modes is also reported as particle size increases.^15^

**Figure 3:**
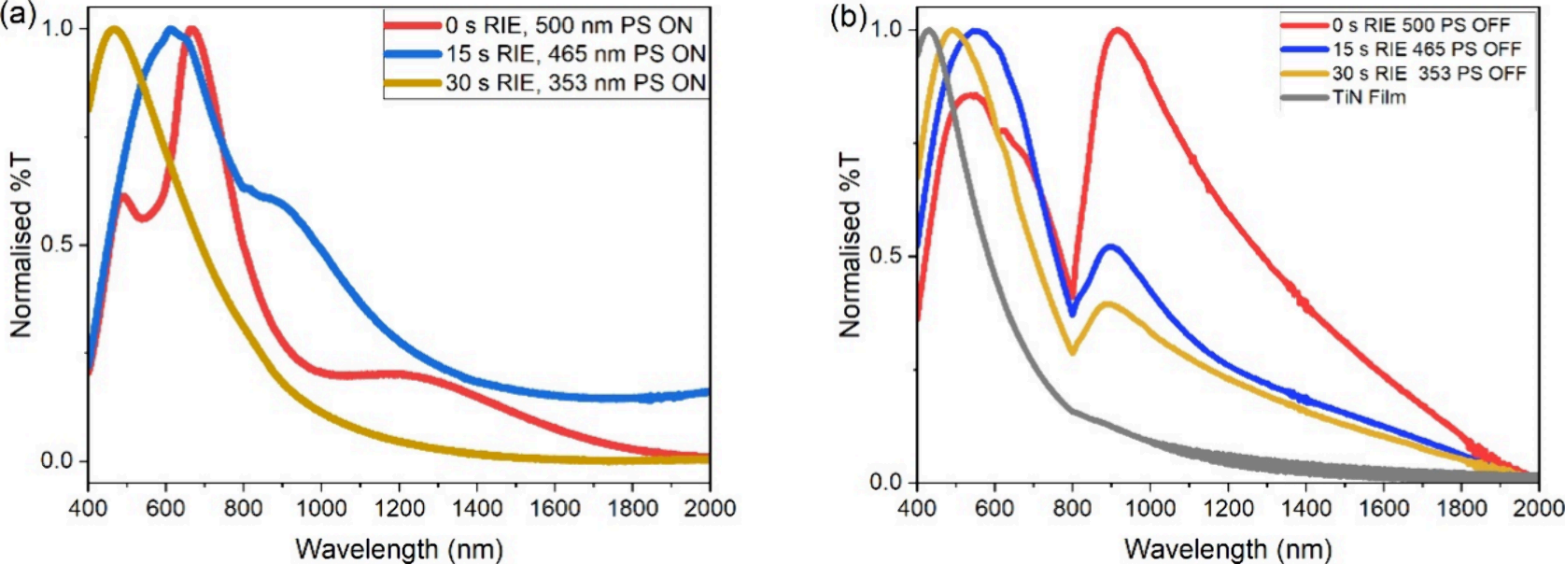
UV–vis-IR transmission spectra for (a) TiN cap-hole arrays and (b) TiN nanotriangles and nanohole arrays.

UV–vis–NIR transmission data collected for the nanotriangle and nanohole arrays produced after mechanical exfoliation of the TiN-coated PS spheres are shown in Figure 3b. The hole diameters are smaller than the PS sphere diameter due to the high mobility of the surface adatoms, yielding deposition underneath the PS mask. The transmission spectra for films deposited on PS without oxygen plasma etching display transmission dips, whereas variations in transmission are less evident in the samples that have been subject to oxygen plasma etching. From the AFM data included in Supplementary Section 3b, the 0 s RIE sample displays the thinnest TiN deposition and the greatest variation in TiN nanofeatures. In contrast, the 15 s and 30 s RIE samples display a more clearly defined nanohole array. This variation in film morphology can provide some explanation for the apparent lack of spectral features in the etched PS samples. Overall, the UV–vis-NIR spectra for TiN hole-cap arrays and TiN nanohole arrays indicate that the optical properties of the nanostructured metamaterial arrays are tunable with feature dimensions.

To compare with the experimental results, we performed finite-difference time-domain (FDTD) simulations (Ansys Lumerical) using a 100 nm-thick TiN film with nanohole arrays. Figure 4a shows a schematic illustration of the TiN nanohole configuration. The TiN film is deposited on a thick glass substrate. Normally incident light with *x*-polarization is used to illuminate the system. To simplify the simulation, the nanoholes are modeled as cylindrical holes with an effective diameter (*d*). We varied the diameters of the cylindrical nanoholes to match the experimental results. Figure 4b presents the simulated transmission spectra for nanohole diameters of 145, 220, and 250 nm. As mentioned above, such smaller hole diameters compared to the PS sphere diameter are due to the high mobility of the surface adatoms yielding deposition underneath the PS mask, which can also be verified by the AFM data in Figures S11 and S12 and SEM images in Figure S8. In contrast to the experimental data presented in Figure 3, simulated spectra were not normalized to the peak value. A comparison of the non-normalized simulated spectra in Figure 4b and experimental spectra from Supplementary Section 3c, Figure S13 indicates that the intensities and spectral features in the simulations are in reasonable agreement.

**Figure 4:**
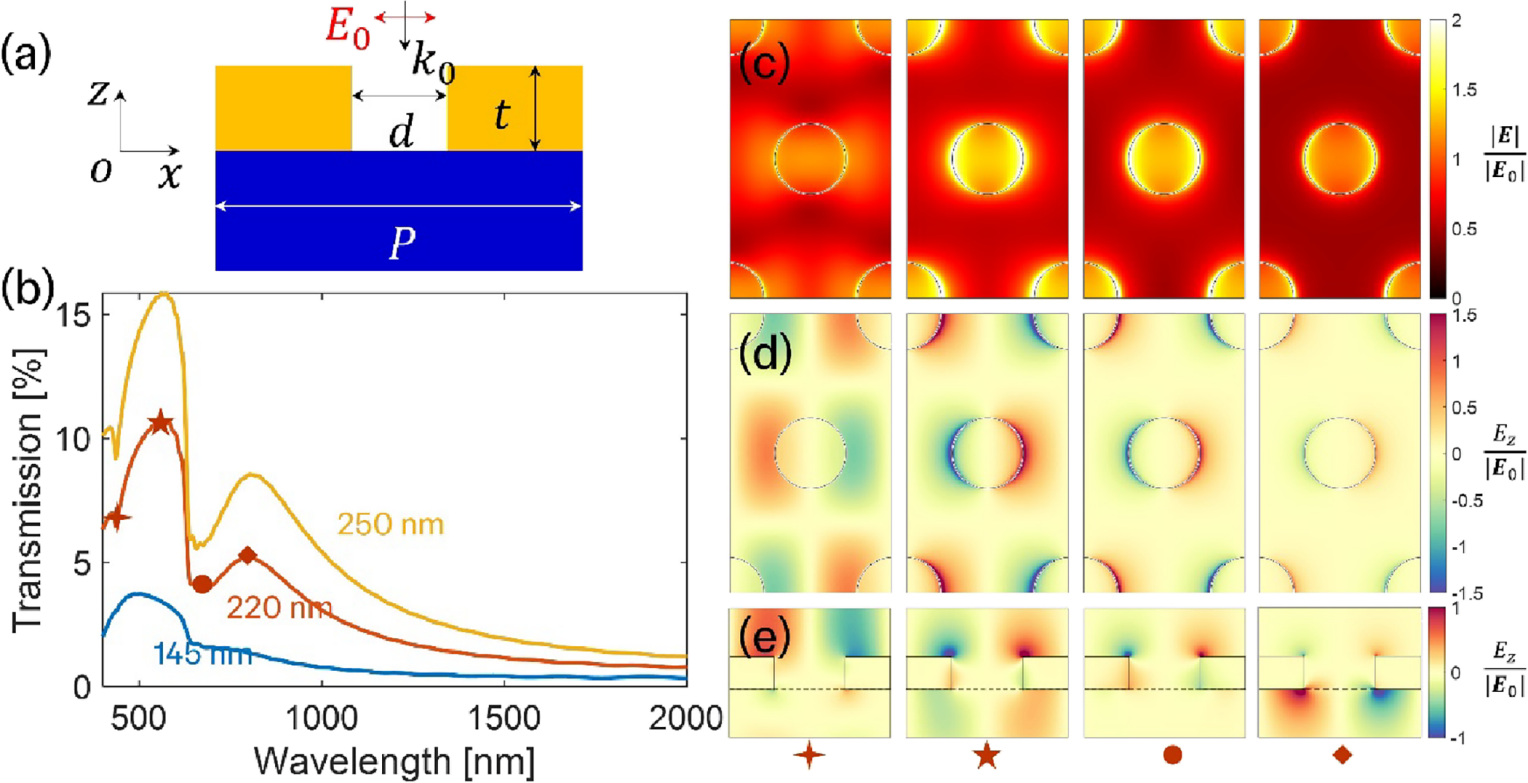
(a) Schematic illustration of TiN nanohole arrays on a silica substrate. The nanoholes form a two-dimensional hexagonal lattice with a side length of 500 nm. The film thickness (*t*) is fixed at 100 nm. The effective cylinder diameter (d) varies across simulations to match the experimental data. (b) Simulated transmission spectra for nanohole of 145, 220, and 250 nm. (c) Electric field amplitude distribution (|*e*|/|*e*_0_|), and (d) the *z*-component of the electric field (e_z/|*e*_0_|), calculated at the corresponding transmission resonance wavelengths. The field is monitored at a plane located 2 nm above the top surface of the TiN film. (e) The *z*-component distribution (*e*_*z*/|*e*_0_|) monitored at a vertical plane passing through the center of the nanohole. The corresponding resonance wavelengths are indicated for each case.

Simulated data are listed in Figure 4. In the simulations, we used material data from Palik^69^ for the glass substrate and employed our measured optical constants for TiN, obtained from a 40 nm-thick film at room temperature. The simulations indicate an increase in the absolute value of transmission with increasing nanohole diameter. This is expected, as the amount of titanium nitride absorbing and scattering light reduces overall. The continuous, unpatterned TiN film has a transmission peak at ∼485 nm concurrent with the ENZ crossover region.

To further understand the resonant modes corresponding to the transmission peaks and valleys, we investigated near-field distributions. Figure 4c,d shows the electric field amplitude (|*e*|/|*e*_0_|) and the *z*-component of the electric field (*e*_*z*_/|*e*_0_|), both calculated at a plane located 2 nm above the TiN film surface. Additionally, Figure 4e presents the *z*-component distribution (*e*_*z*_/|*e*_0_|) monitored at a vertical plane passing through the center of the nanohole. The discrepancies between the simulated and experimental results are primarily attributed to fabrication imperfections.

Such plasmonic arrays fabricated from TiN have previously been reported to have applications in biological and refractive index sensing, enhanced optical absorption and solar driven hydrogen evolution.^11,70–72^ The fabrication process described here is CMOS-compatible and, furthermore, enables the deposition of hole-cap and nanohole arrays onto polymer substrates to facilitate the production of lab-on-a-chip and low-cost sensors. The combined room-temperature HIPIMS deposition and colloidal lithography method described in this work is an imminently scalable method facilitating the fabrication of devices and large area plasmonic arrays and metasurfaces.

## Conclusions

We have reported a method of fabricating nanoscale metamaterial arrays with high plasmonic quality on polymer substrates, enabling low-cost biological sensors and lab-on-chip devices. The method relies on high-power-impulse magnetron sputtering deposition for the room-temperature production of high-quality plasmonic titanium nitride thin films. Films produced using this HIPIMS method are polycrystalline with metallic optical response and can be deposited onto a range of industrially relevant substrates, including polymers. The room-temperature deposition method presented here also enables the fabrication of metamaterial arrays via lift-off, removing the requirement for etching and simplifying the fabrication process. We demonstrated that these metamaterial arrays display a tailorable optical response, enabling them to be adapted to specific applications. The HIPIMS and colloidal lithography methods described in this work are scalable deposition and nanofabrication techniques, and the fabrication method described here has the potential to produce TiN-based plasmonic devices on a scale or over large surface areas.

## Experimental Methods

### Thin Film Deposition

TiN thin films are deposited via high-power impulse magnetron sputtering in a confocal CS-400S cluster system (Von Ardenne Anlagentechnik GmbH), with a target-substrate distance of 90 mm. A minimum chamber base pressure of <3 × 10^–6^ Pa is obtained prior to deposition in each case. Chamber pressure is maintained during deposition using a PID controlled pump throttle valve. The chamber temperature was measured by using a thermocouple positioned 5 mm behind the substrate holder and calibrated for the surface of a Si substrate.

TiN thin films are deposited onto bare glass, steel, Si, PEN, and PI substrates and onto Si, glass, PI, and PEN substrates patterned by using colloidal lithography. Unpatterned substrates are cleaned prior to deposition using a solvent cleaning process consisting of sequential sonication in acetone, isopropyl alcohol (IPA), and water for 10 min each followed by drying with nitrogen. Bare substrates are also plasma-cleaned by inverse sputter etching with Ar to ensure complete removal of surface contaminants before being transported *in vacuo* to the deposition chamber. Patterned substrates are not subject to the same cleaning procedure to maintain the integrity of the colloidal mask pattern.

During deposition, a pure Ti metal target (99.9%, diameter 100 mm) is sputtered in a gas mixture of Ar:N_2_ (gas purity 99.998%) with a ratio of 30:1 at a deposition pressure of 0.3 Pa. A differentially pumped quadrupole mass analyzer (Microvision 2. MKS) was used to monitor residual gas partial pressures *in situ* throughout the deposition process. Example data collected during a TiN deposition are included in Figure S1. The HIPIMS discharge was generated using a Highpulse 4000 G2 generator (Trumpf Hüttinger Elektronik sp. z o.o) operating in constant current mode to apply a peak pulse *I*_pk_ of 45 A, yielding a peak current density of 0.6 A cm^–2^ to the target. The constant current mode of the generator allowed for a stable deposition process with an arc rate of ∼3 arcs/h. A pulse duration of 125 μs was used for each deposition. Example current and voltage data from a typical HIPIMS pulse characteristics are included in Figure S2. No external bias was applied to the substrates during deposition, and substrates remained at floating potential throughout.

In all cases, TiN films are deposited to a total thickness of 100 nm. For depositions onto patterned substrates, a “witness” glass substrate is also included to allow the bare TiN thin film properties to be characterized.

### Colloidal Lithography – Lift-Off

Metamaterial arrays are prepared by depositing TiN onto selected substrates (silicon, glass, polyimide-coated glass, and polyethylene naphthalate) through a polystyrene mask. Substrate masks are prepared via water buffer-based transfer nanosphere lithography or “fishing”. During this process, a hexagonally close-packed (hcp) polystyrene monolayer is prepared at the air–water interface of a vessel containing buffered ultrapure water and then applied to the substrate of choice.

To prepare the hcp monolayer, a 10 wt % solution of PS colloidal particles (Bangs Laboratories Inc., *d* = 320 nm, 500 nm, 620 nm) in a 1:1 mixture with ethanol is first applied to a clean silicon transfer wafer that has been hydrophilized by UV-ozone treatment. Once the PS monolayer has been applied to the Si transfer wafer and is sufficiently dry, the transfer wafer is gradually submerged within a vessel of ultrapure water, pH 8. This releases the monolayer from the transfer wafer to the air–water interface. Repeat transfers yield a large area coverage of hcp PS monolayer, with packing density modified by the pH and addition of a surfactant, sodium dodecyl sulfate (SDS). Transfer of the monolayer to the air–water interface encourages the removal of PS aggregates and bilayers from the solution, thereby improving the quality and uniformity of the PS monolayer.

Prior to fishing, substrates (Si, glass) are first cleaned using the same process described above and then hydrophilized by exposing to a UV-ozone environment in a UV-ozone cleaner (Ossila) for a minimum of 15 min. The hcp PS mask is then transferred from the water–air interface onto the hydrophilic substate surface. Samples are dried in air before subsequent characterization and thin film deposition. Note, for PI and PEN substrates, the cleaning and hydrophilization steps are omitted and PS is applied directly to the substrate. This is to mitigate any damage to the polymer surfaces.

Following application of the PS mask to the desired substrates, it is possible to modify the diameter of the PS colloids and therefore alter the packing density and dimensions of the nanofeatures. This is achieved using reactive ion etching (RIE). The PS colloids are exposed to an oxygen plasma, and the PS is etched, resulting in a reduction of diameter and an increase in spacing between the individual colloidal mask particles. An RIE system (Sentech Etchlab 200) was used to create an oxygen plasma with an applied power of 100 W, 20 Pa of O_2_. Varied etch times (0, 15, 30, and 60 s) were used to produce PS masks with different diameters and spacing. Once the PS masks were prepared, the masked substrates were coated with TiN thin films by using the room-temperature deposition method described above.

### Characterization

The dielectric permittivity of TiN thin films was extracted from spectroscopic ellipsometry data collected using a J.A. Woollam V-VASE Spectroscopic ellipsometer. Ellipsometric parameters psi and delta were measured at incident angles of 65, 70, and 75° over the spectral range 300–1600 nm. The optical properties of the films were extracted from the experimental data by fitting them to a Drude-Lorentz model consisting of one Drude and two Lorentz oscillators. The fit was optimized by minimizing the mean squared error (MSE) using the Marquardt minimization algorithm. Optical absorbance and transmission data were collected for nanopatterned arrays using a Cary 5000 UV–vis spectrometer. Spectra were corrected with reference to an air background and data were normalized to the peak transmission values measured for each sample.

Atomic force microscopy (AFM) micrographs were collected using an Oxford Instruments MFP-3D Origin+ Asylum Research AFM. The AFM operated in AC Air topography mode and SCOUT 70 tips. Scanning electron micrographs were collected using a Gemini 1 Zeiss Sigma 300 field emission SEM operating with an accelerating voltage of 5 keV. Thin film X-ray diffraction data are collected using a Malvern Analytical Empyrean MultiCore high-performance X-ray diffractometer operating in θ–2θ geometry. The system uses a monochromated Cu Kα source with a wavelength of 1.54 Å and is equipped with a 2D PIXcel detector.

## Supplementary Material



## Notes

Thin film depositions parameters: example HIPIMS discharge, residual gas analyzer data and plasma characterization; thin film characterization: XRD, optical figures of merit; nanofabrication method schematic and nanoarray characterization including SEM, AFM and UV–vis-IR data; witness sample characterization: XRD and ellipsometry (PDF)
