# Supplementary material for: TiN Plasmonic Metamaterial Arrays Fabricated at Low Temperatures on Versatile Substrates
Source: ACS Appl Opt Mater. 2025 Dec 8;3(12):2883–92. doi: 10.1021/acsaom.5c00449 (PMC12751110; doi:10.1021/acsaom.5c00449)
Supplement: Supplementary file 1 [file ot5c00449_si_001.pdf]

TiN plasmonic metamaterial arrays fabricated at low temperatures on versatile substrates

Supporting Information

Ryan Bower[1*], Daniel A L Loch[3], Ethan Muir[3], Bruno Rente[1], Xiaofei Xiao,[2] Ming Fu[2], Papken Eh. Hovsepian[3], Arutiun P. Ehiasarian[3], Rupert Oulton[2], Peter K. Petrov[1]

1. *Department of Materials, Royal School of Mines, Imperial College London, Exhibition Road, South Kensington, London SW7 2AZ, UK*
2. *Department of Physics, Blackett Laboratory, Imperial College London, Prince Consort Road, South Kensington, London SW7 2BW, UK*
3. *National HIPIMS Technology Centre, Materials and Engineering Research Institute, Sheffield Hallam University, Howard Street, Sheffield S1 1WB, UK*

* *Corresponding author email: r.bower16@imperial.ac.uk*

Contents

1. Thin film deposition parameters 2
2. Thin film characterisation 4
   a. Structural characterisation 4
   b. Optical characterisation: literature comparison 5
3. Nanostructure preparation and characterisation 6
   a. SEM 6
   b. AFM 8
   c. UV-Vis-IR data 11
4. Witness Sample Characterisation 12

1. Thin film deposition parameters

HIPIMS pulse characteristics were monitored throughout the deposition process to ensure a consistent deposition. For all depositions, the HIPIMS discharge was operated in constant current mode, with a peak current (I_{pk}) of 45 A, and a pulse duration of 120 µs. An example HIPIMS current and voltage profile is included in Figure S 1.

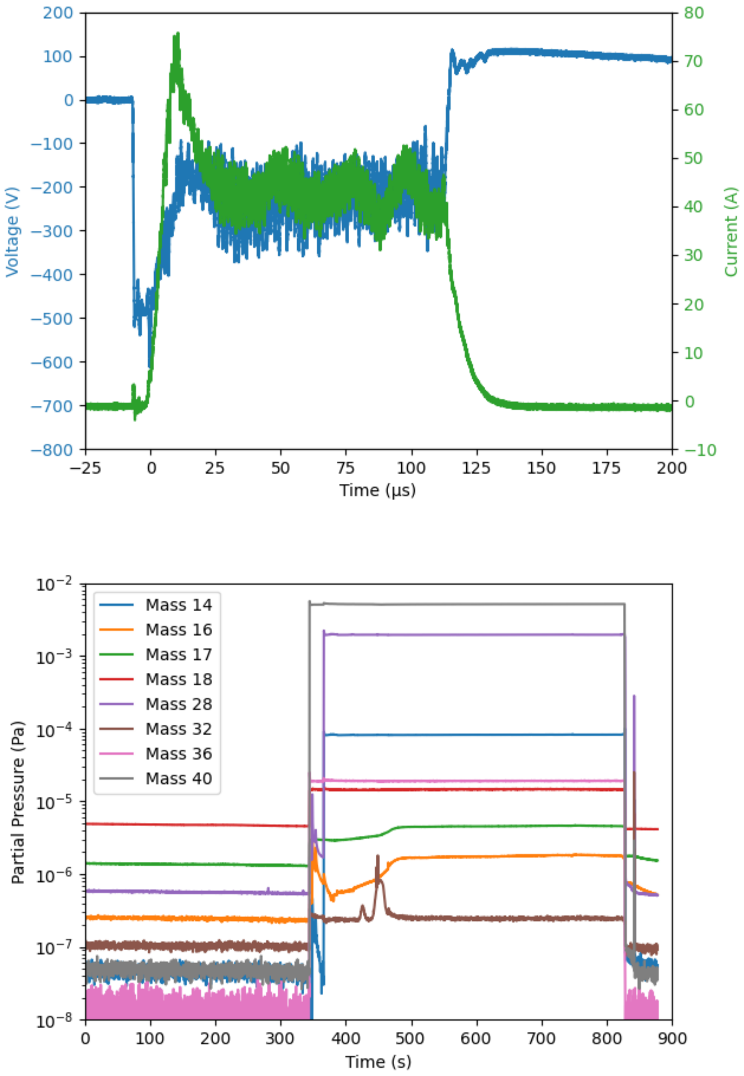

Figure S 1: (Left) Current and voltage plot for a typical HIPIMS pulse collected during TiN deposition. (Right) RGA data collected for the duration of a TiN deposition.

In addition to monitoring the HIPIMS voltage pulse, residual gas analysis was used to assess the gas composition within the deposition chamber during the deposition. As demonstrated in Figure S 1, the partial pressure of process gases nitrogen (mass 14, mass 28) and Ar (mass 40) is constant throughout the deposition. The presence of gases corresponding to masses of 16, 17, 18 and 32 arise due to the presence of water and oxygen within the process chamber and contaminants in the process gas bottle.

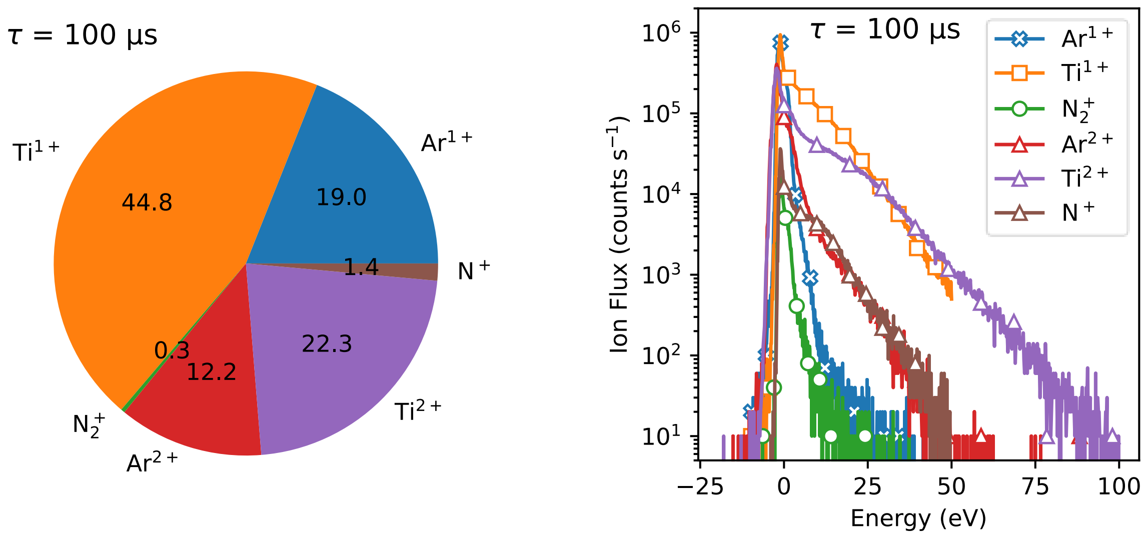

Figure S 2: Characteristics of the ion flux arriving to the substrates for a typical HIPIMS pulse with duration τ = 100 µs collected during TiN deposition. (Left) Composition (Right) Ion energy distribution function.

The characteristics of the ion flux arriving at the substrates were measured in a dedicated experiment where the substrate holder was replaced with a mass spectrometer mounted on a specially designed flange. A PSM003 energy-resolved mass spectrometer (Hiden Analytical Ltd., UK) was used to record the time-averaged chemical composition and energy distribution function of ions arriving at the substrate.

As demonstrated in Figure S 2, the flux comprised predominantly metal ions with significant fraction being double ionised (Ti^{2+}). The gas fraction comprised predominantly Ar^{1+} and Ar^{2+} ions. Approximately 35% of ions were double-charged, which would accelerate them to double the energy across the sheath even without biasing the substrates. Nitrogen ions were mostly in a dissociated state with N^{1+} : N_2^+ ratio of 4.7. The energies of metal ions and dissociated nitrogen N^{1+}, shown in Fig. S2 was significantly greater than those of N_2^+ and Ar^{1+}, and was gained from the sputtering cascade responsible for ejecting them from the target. The latter (gas) ions gained a small fraction of their energy from the sputtering cascade as indicated by a high-energy shoulder in the energy distribution function.

2. Thin film characterisation

a. Structural characterisation

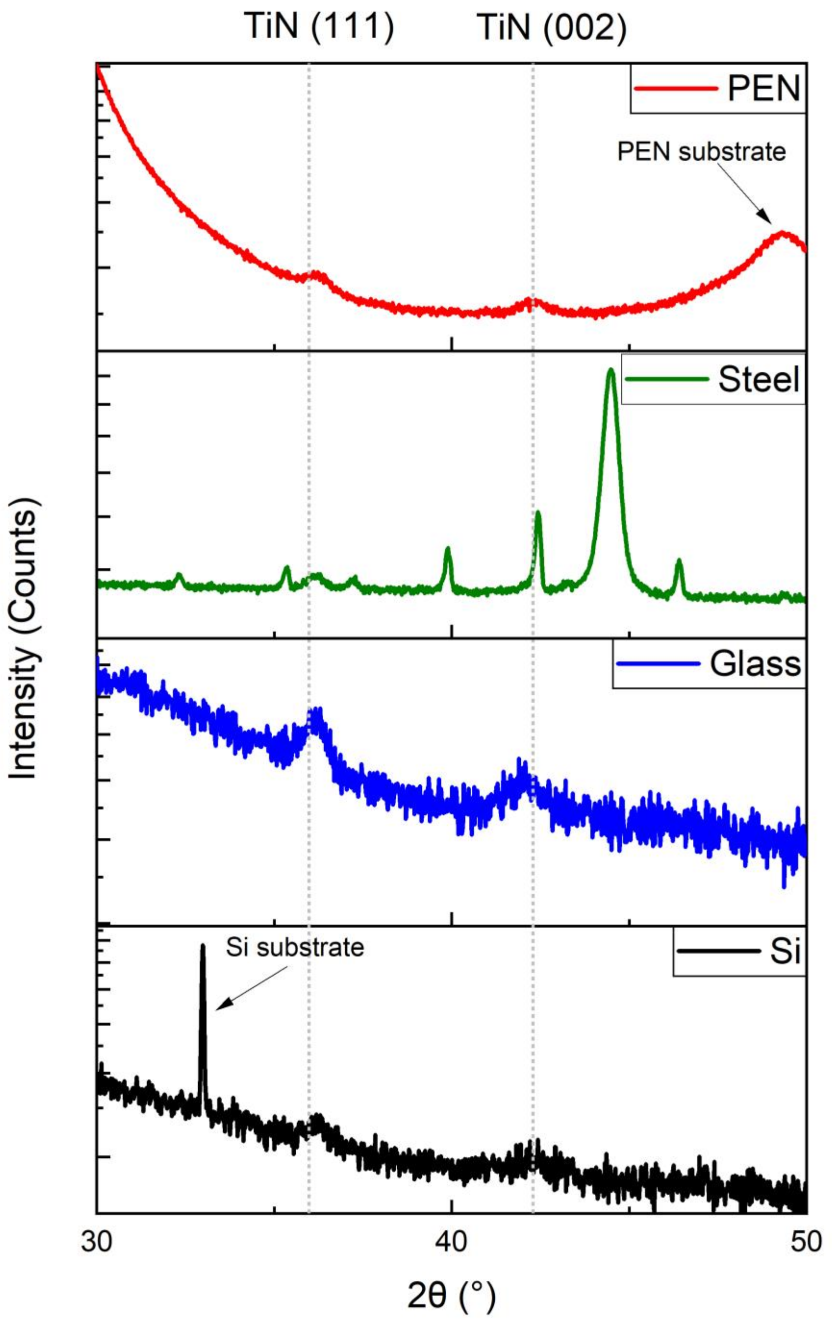

Figure S 3: XRD data for TiN thin films deposited by HIPIMS at room temperature on various substrates.

**Substrate**	**111 position (° 2θ)**	**002 position (° 2θ)**	**Lattice parameter (Å)**
PEN	36.18	42.25	4.55
Steel	36.14	-	4.52
Glass	36.14	42.04	4.56
Silicon	36.20	42.3	4.55

b. Optical characterisation comparison

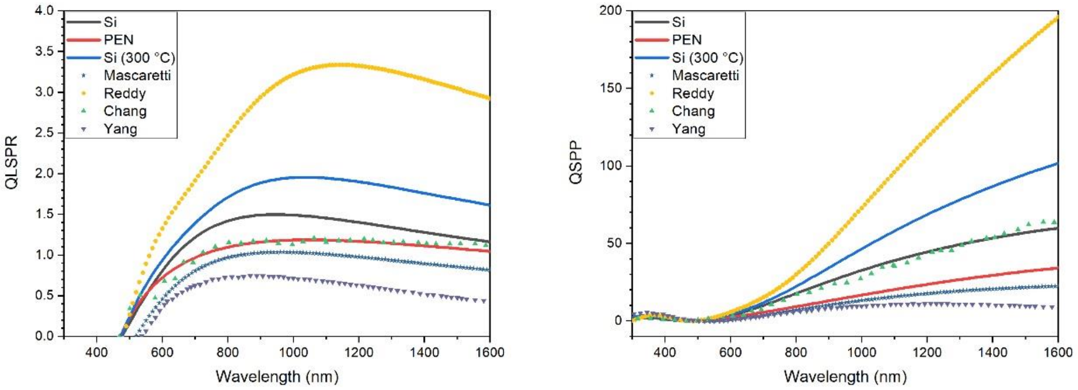

Figure S 4: Comparison of Q_{SPP} and Q_{LSPR} for TiN thin films reported in this work with a selection of TiN films reported in the literature.

A comparison of plasmonic figures of merit for the films reported in this work with some films reported in literature. TiN films reported by Reddy et al[1], Mascaretti et al,[2] Chang et al[3] and Yang et al[4] were deposited using RF magnetron sputtering at 850 °C on sapphire; pulsed DC deposition on Si at room temperature; RF magnetron sputtering at room temperature on Si with a short substrate target distance and using HIPMS with peak power density of 75 W cm^{-2} at room temperature onto glass substrates.

Further comparison of TiN films with other literature data can be found in the topical review by Mascaretti et al[5] and in the work of Patsalas et al[6] and Chang et al, for example.[3]

(1) Reddy, H.; Guler, U.; Kudyshev, Z.; Kildishev, A. V.; Shalaev, V. M.; Boltasseva, A. Temperature-Dependent Optical Properties of Plasmonic Titanium Nitride Thin Films. *ACS Photonics* **2017**, *4* (6), 1413–1420. https://doi.org/10.1021/acsphotonics.7b00127.

(2) Mascaretti, L.; Barman, T.; Bricchi, B. R.; Münz, F.; Li Bassi, A.; Kment, Š.; Naldoni, A. Controlling the Plasmonic Properties of Titanium Nitride Thin Films by Radiofrequency Substrate Biasing in Magnetron Sputtering. *Appl Surf Sci* **2021**, *554*, 149543. https://doi.org/10.1016/J.APSUSC.2021.149543.

(3) Chang, C.-C. C.; Nogan, J.; Yang, Z.-P. P.; Kort-Kamp, W. J. M. M.; Ross, W.; Luk, T. S.; Dalvit, D. A. R. R.; Azad, A. K.; Chen, H.-T. T. Highly Plasmonic Titanium Nitride by Room-Temperature Sputtering. *Sci Rep* **2019**, *9* (1). https://doi.org/10.1038/s41598-019-51236-3.

(4) Yang, Z.-Y.; Chen, Y.-H.; Liao, B.-H.; Chen, K.-P. Room Temperature Fabrication of Titanium Nitride Thin Films as Plasmonic Materials by High-Power Impulse Magnetron Sputtering. *Opt Mater Express* **2016**, *6* (2), 540. https://doi.org/10.1364/ome.6.000540.

(5) Mascaretti, L.; Mancarella, C.; Afshar, M.; Kment, Š.; Bassi, A. L.; Naldoni, A. Plasmonic Titanium Nitride Nanomaterials Prepared by Physical Vapor Deposition Methods. *Nanotechnology* **2023**, *34* (50), 502003. https://doi.org/10.1088/1361-6528/ACFC4F.

(6) Patsalas, P.; Kalfagiannis, N.; Kassavetis, S. Optical Properties and Plasmonic Performance of Titanium Nitride. *Materials* **2015**, *8* (6), 3128–3154. https://doi.org/10.3390/ma8063128.

3. Nanostructure preparation and characterisation

TiN was deposited through a self-assembled colloidal mask with initial PS sphere diameter of 500 nm. A schematic of the colloidal lithography process is included below:

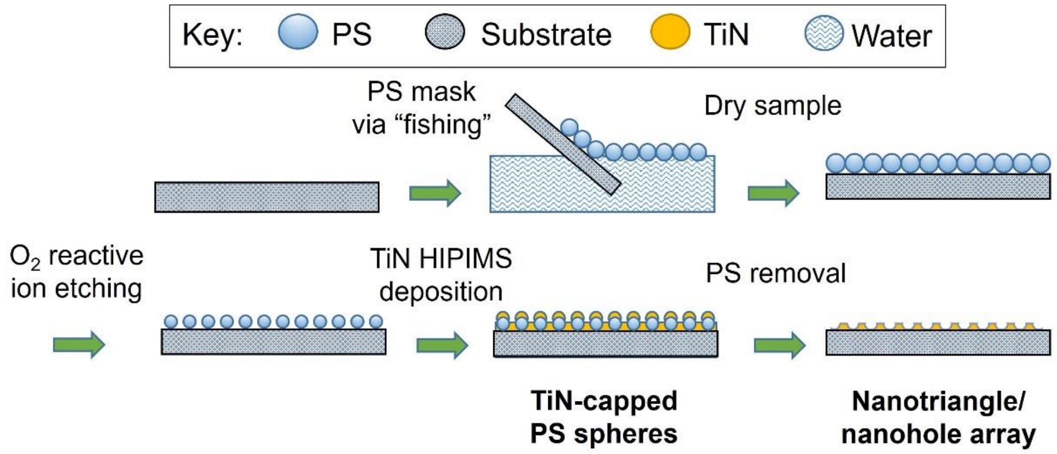

Figure S 5: Schematic of colloidal lithography fabrication process for TiN nanofeatures.

a. SEM

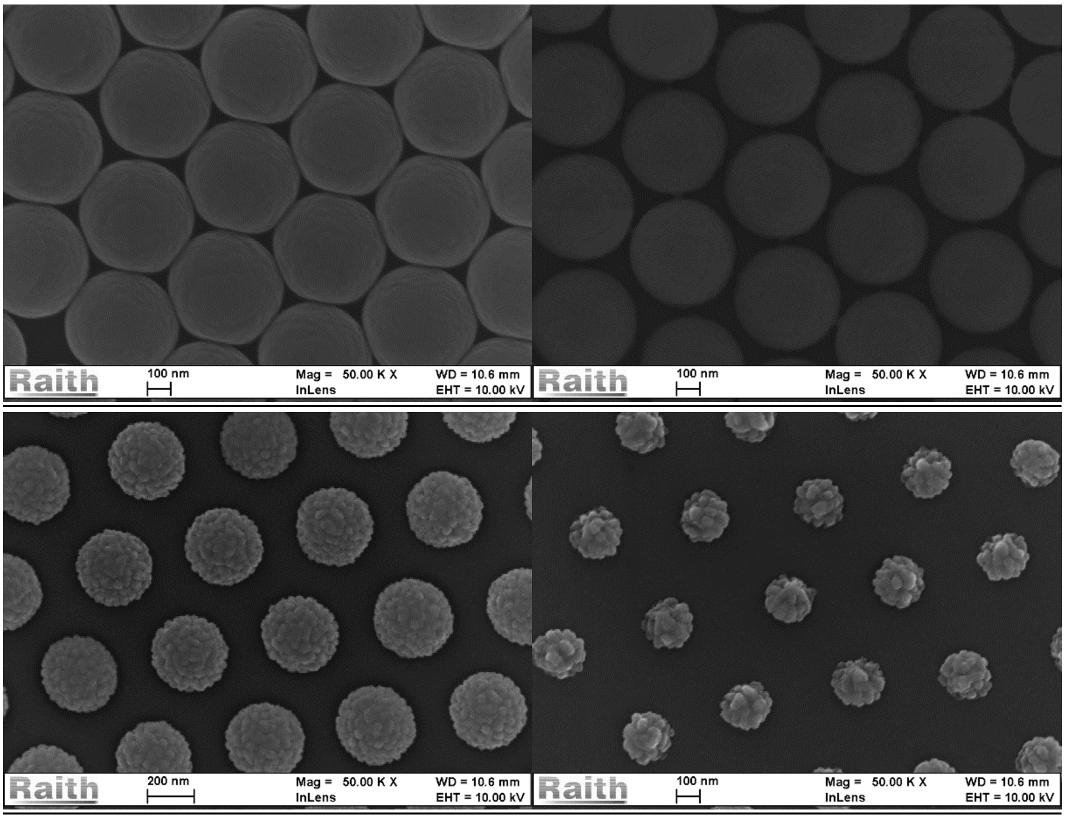

Figure S 6: SEM micrographs of TiN coated PS spheres on Si substrates after 0s, 15s, 30s, and 60s O_2 RIE.

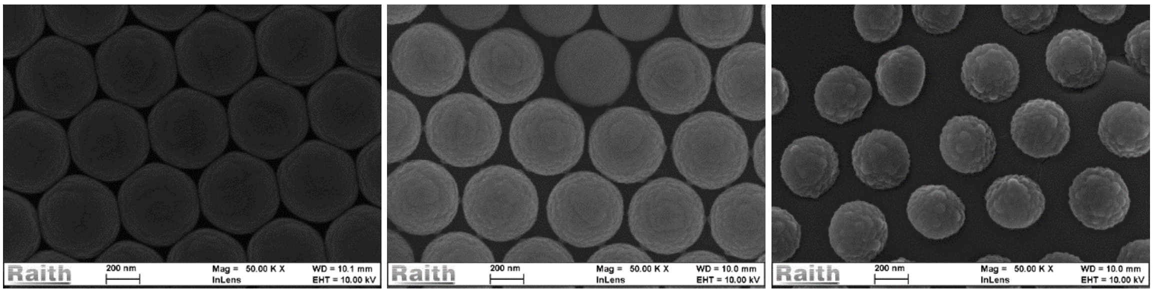

Figure S 7: SEM micrographs of TiN coated PS spheres on glass substrates after 0s, 15s, and 30s O_2 RIE.

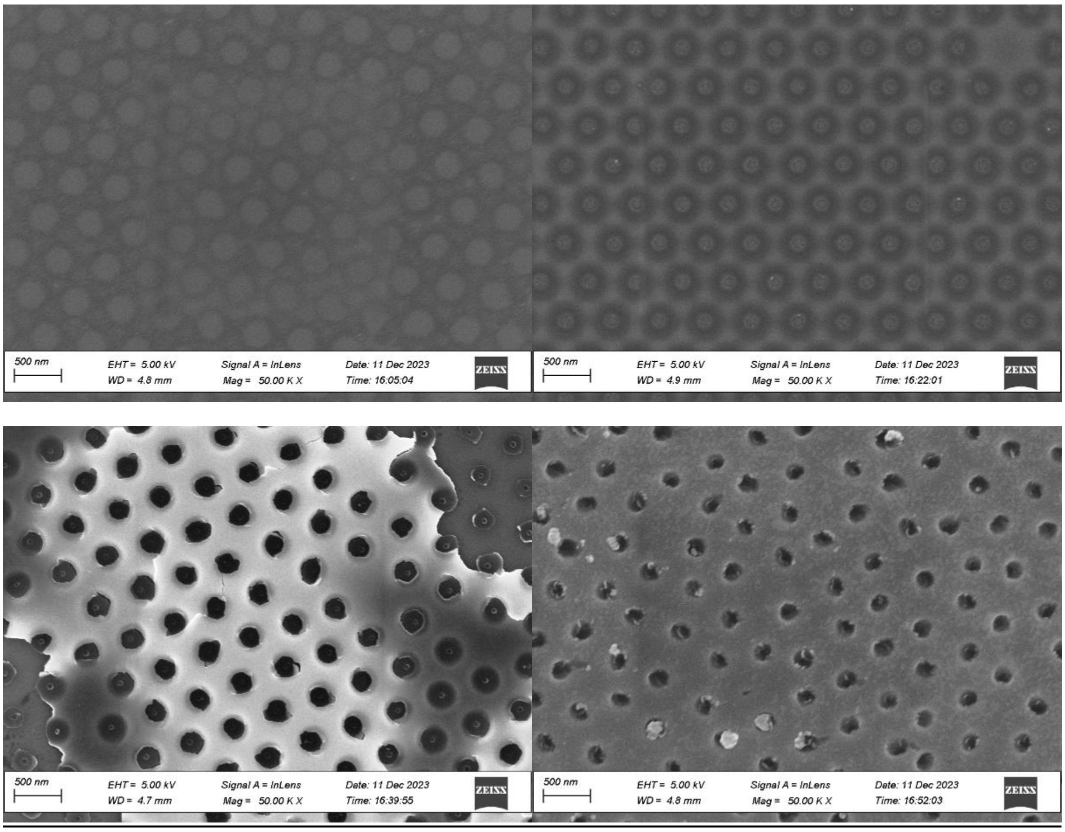

Figure S 8: SEM micrographs of TiN nanohole arrays on Si substrates prepared after 0s, 15s, 30s, and 60s O_2 RIE, thin film deposition and lift-off of PS colloidal mask.

b. AFM

PS ON	Si	Si 3D
0 s	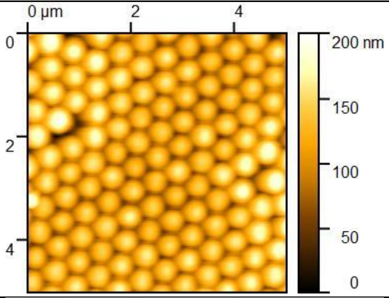	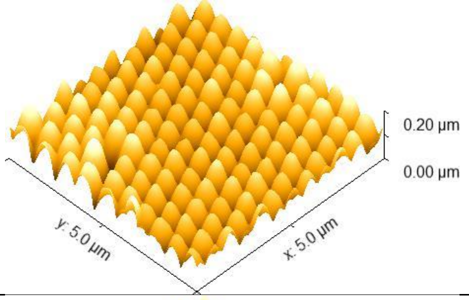
15 s	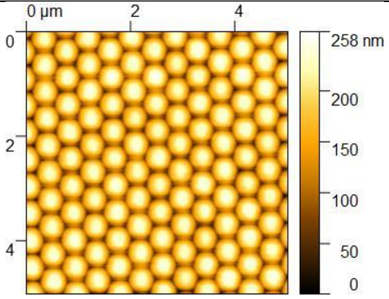	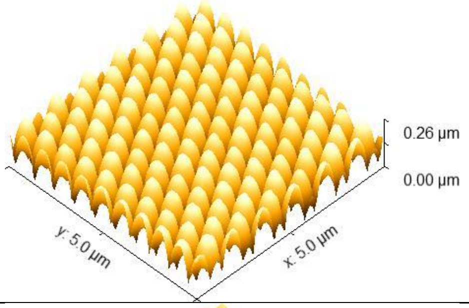
30 s	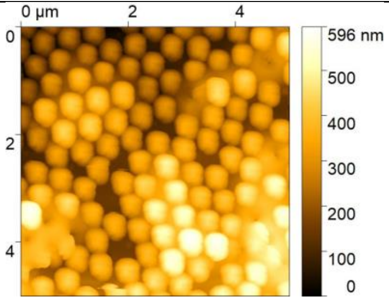	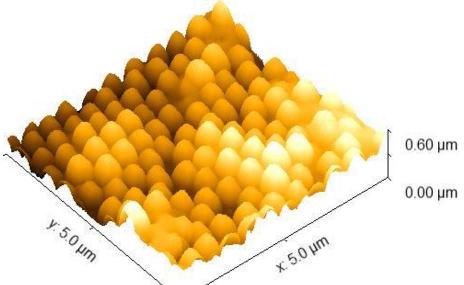
60 s	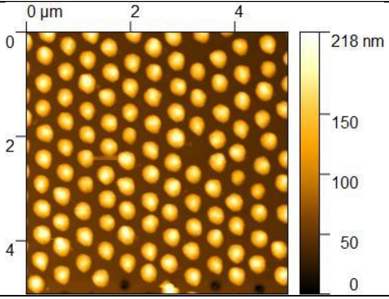	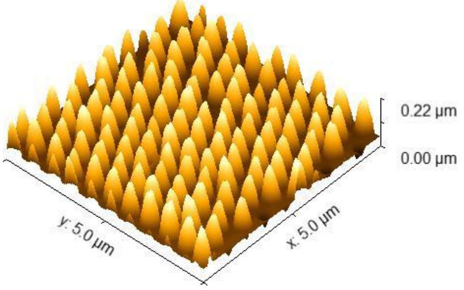

Figure S 9: AFM micrographs for TiN-coated PS masks on Si substrates

	Glass	Glass (3D)
0 s	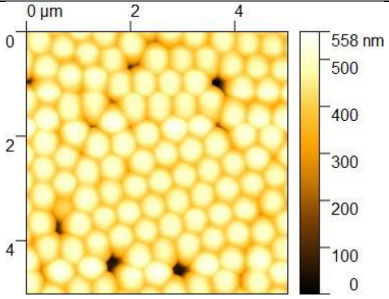	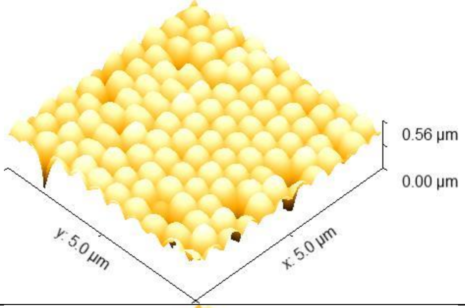
15 s	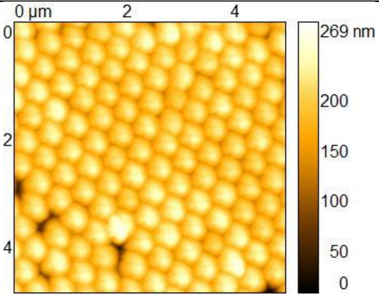	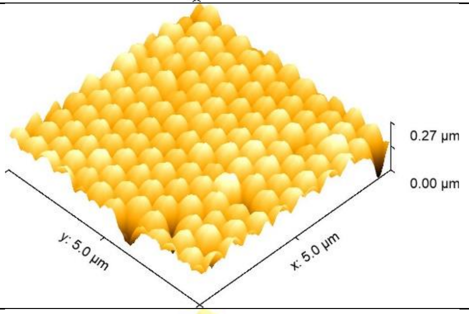
30 s	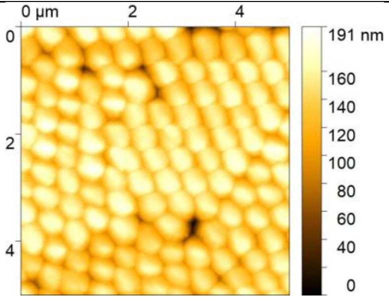	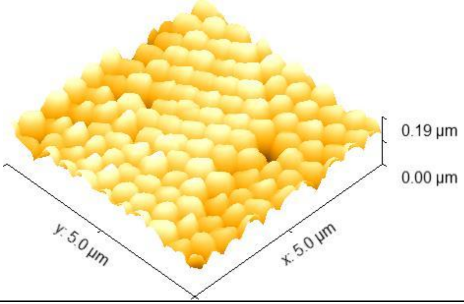

Figure S 10: AFM micrographs for TiN-coated PS masks on glass substrates

PS OFF	Si	Si 3D	Line profiles
0 s	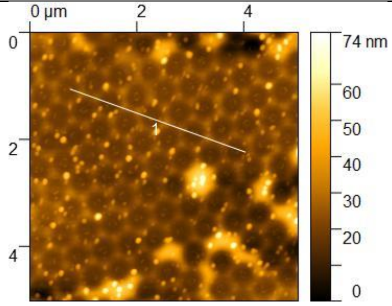	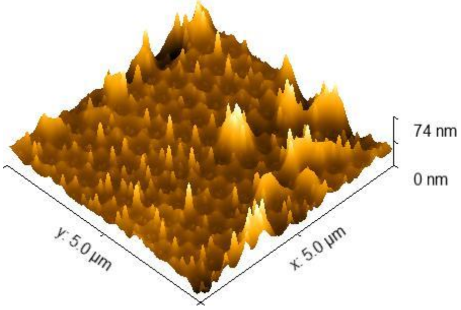	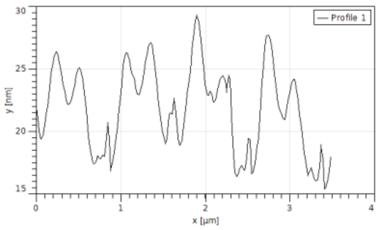
15 s	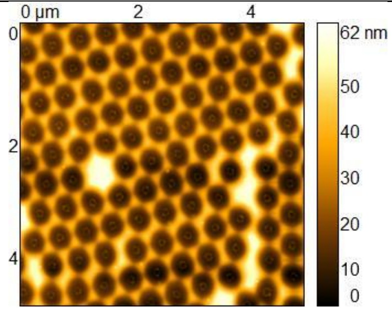	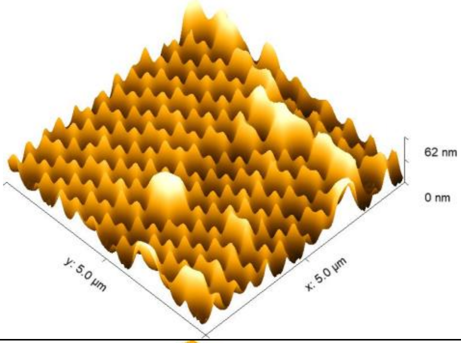	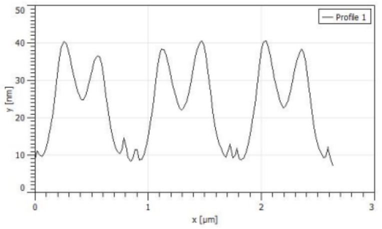
30 s	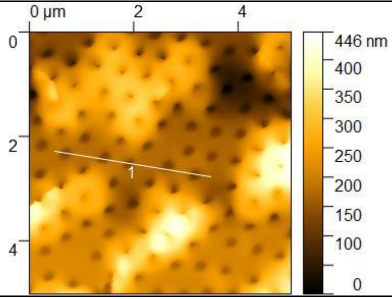	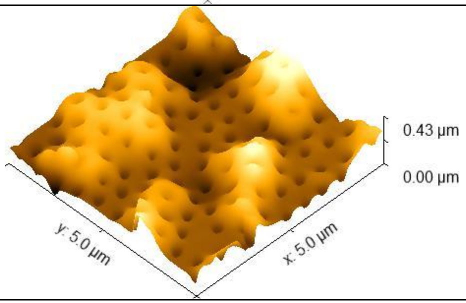	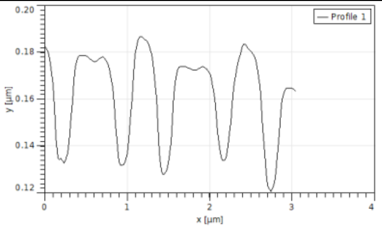

Figure S 11: AFM micrographs and line profiles for TiN nano-triangle and nano-hole arrays on Si substrates

PS OFF	Glass	Glass (3D)	Profile
0 s	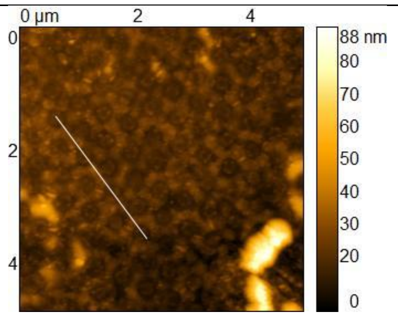	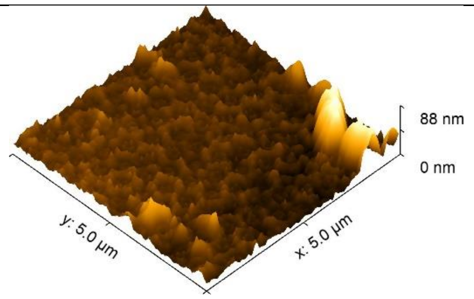	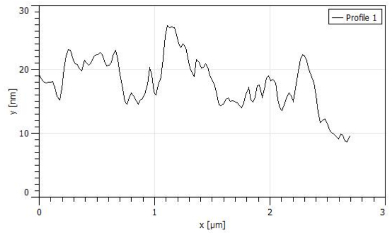
15 s	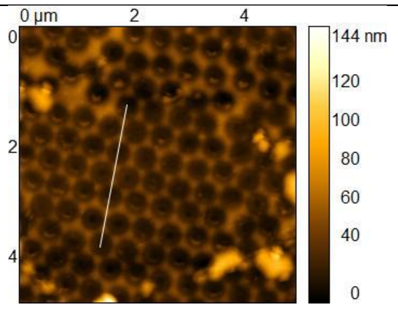	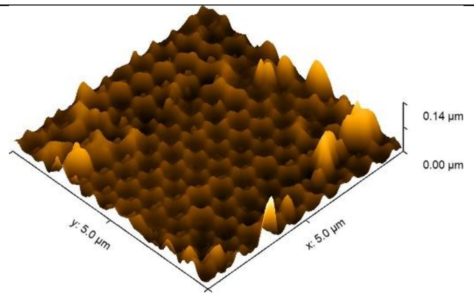	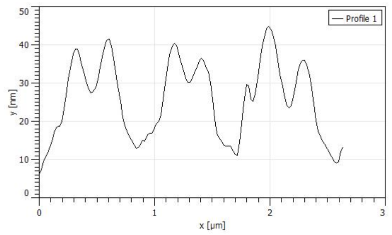
30 s	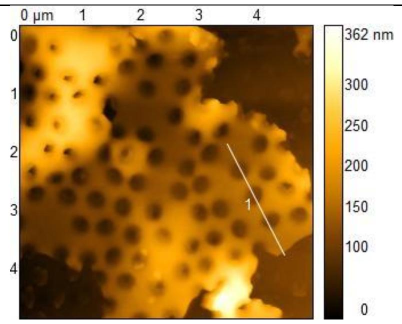	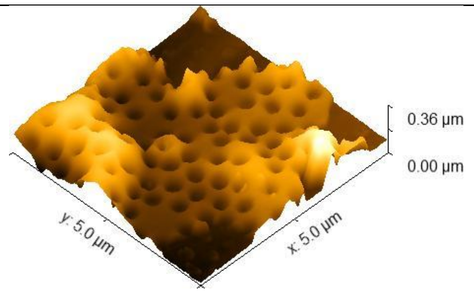	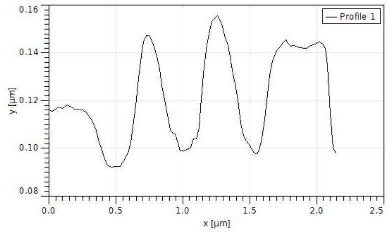

Figure S 12: AFM micrographs and line profiles for TiN nano-triangle and nano-hole arrays on glass substrates

c. UV-Vis-IR data

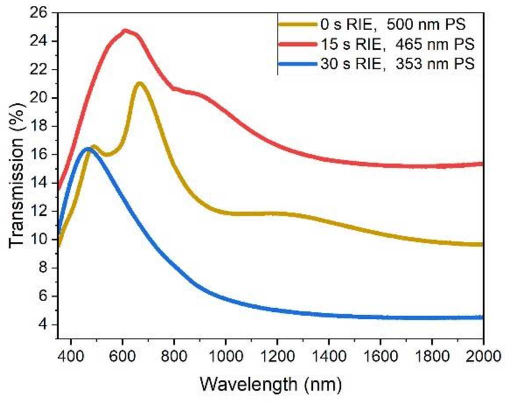

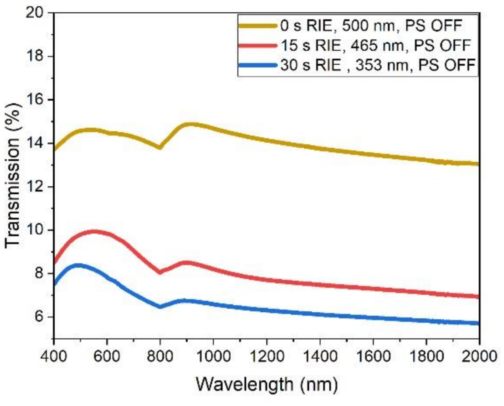

Figure S 13: UV-vis-IR transmission spectra for TiN cap-hole arrays (left) and TiN nanotriangle and nanohole arrays (right). Note, data is not normalised to peak values.

4. Witness Sample Characterisation

When depositing TiN onto patterned substrates coated with a polymer mask, a blank “witness” sample was included in every deposition run to allow the film quality to be assessed and to identify any issues with film quality. A total of 8 room temperature depositions of TiN were completed, each with the same deposition parameters, as summarised above.

Real and imaginary dielectric permittivity data for the witness samples are included in Figure S 14. Qualitatively, the films display consistent stoichiometry, as indicated by the lack of variation in the crossover frequency of the real permittivity. The imaginary permittivity is also consistent for all films, indicating little variation in the optical losses. Minor differences in the magnitude of the dielectric permittivity are observed at near-IR wavelengths potentially arising due to variations in grains size and interface effects.

X-ray diffraction data were also collected for the witness samples, Figure S 15. The diffraction data display the presence of TiN 111 and 002 peaks, with preferential texture in the 111 crystallographic directions.

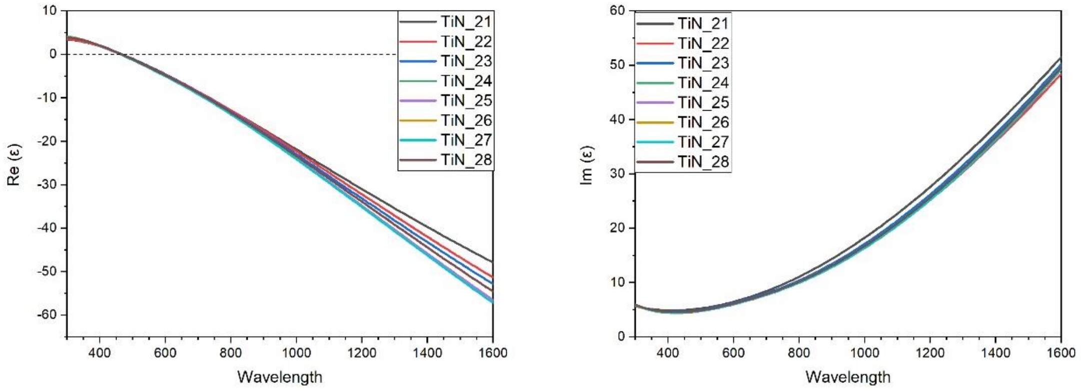

Figure S 14: Real and imaginary dielectric permittivity of “witness” TiN thin films deposited at room temperature onto glass substrates. Permittivity data is extracted from spectroscopic ellipsometry measurements.

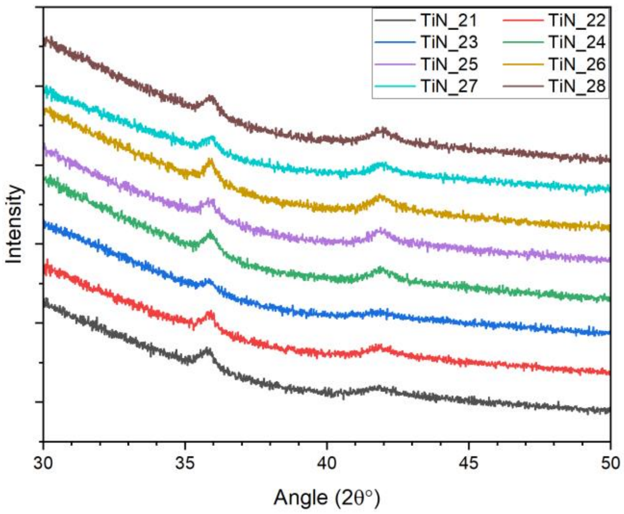

Figure S 15: XRD data for glass witness samples.
